# Anatomical, functional, and blood-born predictors of severity of brachycephalic obstructive airway syndrome severity in French Bulldogs

**DOI:** 10.3389/fvets.2024.1486440

**Published:** 2025-01-09

**Authors:** Claudia Schmid, Aline R. Steiner, Léonie Spielhofer, Meltem Galfetti, Nikki Rentsch, Nikolay Bogdanov, Johannes Vogel, Regina Hofmann-Lehmann, Sonja Hartnack, Georgy Astakhov, Reinhard Furrer, Anna Bogdanova, Iris Margaret Reichler

**Affiliations:** ^1^Red Blood Cell Research Group, Institute of Veterinary Physiology, Vetsuisse Faculty, University of Zurich, Zurich, Switzerland; ^2^Clinic of Reproductive Medicine, Vetsuisse Faculty, University of Zurich, Zurich, Switzerland; ^3^Tierarztpraxis Lauenen, Thun, Switzerland; ^4^Kleintierpraxis Seegarten AG, Schmerikon, Switzerland; ^5^Bessy’s Kleintierklinik, Regensdorf-Watt, Switzerland; ^6^Clinical Laboratory, Department for Clinical Diagnostics and Services, Vetsuisse Faculty, University of Zurich, Zurich, Switzerland; ^7^Center for Clinical Studies, Vetsuisse Faculty, University of Zurich, Zurich, Switzerland; ^8^Section of Epidemiology, Vetsuisse Faculty, University of Zurich, Zurich, Switzerland; ^9^Applied Statistics Group, Department of Mathematical Modeling and Machine Learning, Faculty of Science, University of Zurich, Zurich, Switzerland

**Keywords:** brachycephaly, brachycephalic obstructive airway syndrome, severity grading, trotting test, anatomical conformation, whole body plethysmography, carboxyhemoglobin

## Abstract

Brachycephalic breeds suffer from respiratory distress known as brachycephalic obstructive airway syndrome (BOAS) and the multiple comorbidities associated with it. Targeted breeding toward a more BOAS-free phenotype requires accurate and least invasive detection of BOAS severity grades that are accessible and accepted by the breeders and kennel clubs. This study aimed to compare the-outcome of morphometric anatomical examination with functional tests such as exercise tests and plethysmography for the detection of BOAS severity in a group of 84 French Bulldogs. In addition, we investigated the possibility of assessing the severity of BOAS using blood parameters that were found to vary between the brachycephalic and non-brachycephalic dogs in our previous study. We found the results of the trotting test to be most reliable compared to the outcome of respiratory performance assessment using plethysmography. Of all the candidate blood parameters tested, carboxyhemoglobin and oxyhemoglobin levels were the most predictive as on-side but not self-standing indicators of BOAS severity grade. Aggravation of BOAS manifestation was associated with mild stress erythropoiesis and oxidative stress. Based on our findings, we suggest continuing to use the trotting test as the method of choice for the selection of breeding dogs; in questionable cases, a temperature increase of more than 0.4°C indicates at least grade 2. Furthermore, co-oximetry could be used as an additional test to enable assignment to one of the two BOAS grades in dispute. Among the limitations of this study are the focus on one breed and the low number of animals with severe clinical signs of BOAS in the study cohort.

## 1 Introduction

Brachycephalic breeds are becoming increasingly popular worldwide. According to the American Kennel Club rankings, the French Bulldog breed moved from position 12 in January 2012 to the first ranking place in 2022. The AMICUS database reports a population of 8,429 French Bulldogs registered in Switzerland in January 2016 expanding to 16,015 in January 2023.

The most obvious anatomy-related disease in brachycephalic dogs is brachycephalic obstructive airway syndrome (BOAS) (1). Narrowing of the upper respiratory tract due to stenotic nares, aberrant nasal turbinates, tracheal hypoplasia and soft palate elongation increases upper airway resistance (2). Furthermore, the relative macroglossia in brachycephalic breeds may contribute to upper airway obstruction (3). Under exercise or at higher ambient temperature, the negative pressure required to overcome this resistance causes swelling and inflammation of the soft tissue, which in turn aggravates the clinical signs (4, 5). Typical clinical signs are respiratory noises, exercise intolerance and dyspnea (6). Another direct consequence of the shortened muzzle is a smaller mucosal area available for thermoregulation in the upper airway respiratory tract. As the upper respiratory tract is essential for regulating body temperature in dogs (7), brachycephalic dogs are more likely to suffer from hyperthermia. BOAS may cause not only life-threatening respiratory distress and hyperthermia, but also seems to affect the gastrointestinal (8–10) and the cardiovascular function (11).

The above-mentioned numerous disorders of brachycephalic dogs are presumably associated with the shortened skull (12). Brachycephalic cranial anatomy is associated with the neuroanatomical changes resulting in specific patterns of the sleep-recorded EEGs, impaired circulation and reduced absorption of CSF, as well as white matter loss as reviewed in Iotchev et al. (13). These changes pose health risks, poor quality sleep (14) and may limit brain function as well as activity in brachycephalic dogs. As a result, the life expectancy of brachycephalic breeds was on average 9.8 years compared to 11.5 years in dolichocephalic and 11.9 years in mesocephalic dogs (15). When listing the life expectancy of the 50 most common dog breeds in Switzerland, the French Bulldog comes off worst with just 7.7 years (15). This is particularly alarming given the negative correlation between body size and life expectancy.

The choice of radical to modest solutions aiming to reduce the suffering and improve the quality of life of brachycephalic dogs varies between European countries. In Norway, for example, the breeding of King Charles Spaniels is generally not banned, but certain requirements should be met before breeding. Several other countries including Switzerland have chosen to control the breeding of brachycephalic dogs to reduce the numbers of severely affected parents and offspring. The Swiss Animal Welfare Act (Schweizer Tierschutzgesetz) stratifies the dogs into four severity grades depending on interference with physical performance and anatomical features of the dogs: from 0 with no health issues associated with BOAS to 3 with life-threatening clinical signs of BOAS. Breeding is allowed with animals of strain grade 0 or 1. An animal of severity grade 2 may be bred only with an animal of BOAS grade 0 or 1. The breeding objective is to ensure that the grade of the offspring is lower than that of the parents, i.e., progeny testing is mandatory. Dogs with BOAS severity grade 3 do not enter the breeding program (16).

Until now, the methods used for diagnosing and grading BOAS are based on several anatomical or functional approaches. Anatomical approaches range from soft-tape and visual nostril assessments (17) to computer tomography (CT) (18, 19) and endoscopy (19). While soft-tape measurements are non-invasive and easy to obtain, they suffer moderate to poor inter-rater reliability (17). Among externally accessible anatomical parameters, stenotic nostrils have been one of the most robust predictors of BOAS severity across different breeds, including French Bulldogs (17). However, nostril assessment alone does not cover the full range of anatomic anomalies in brachycephalic dogs. Attempts of combining soft tape measurements with nostril assessment, body condition score, and control variables of age, sex, and neuter status in breed-specific multivariate logistic regression models resulted in 37% (French Bulldogs, English Bulldogs) to 48% (Pugs) explained variance in the binary distinction of BOAS-affected versus-non-affected dogs (17). In contrast to these less precise and investigator-dependent anatomical test, CT (18, 19) and endoscopy (19) allow a detailed assessment of relevant anatomical structures. Considering the increased risk of anesthesia in brachycephalic dogs (20), CT and endoscopy seem to be too invasive for screening purposes.

Functional approaches typically use variations of exercise tests (ET). Dogs are exercised for a fixed time or distance, and based on clinical assessment of respiratory noise, respiratory effort and/or recovery times, are assigned one of four BOAS grades (6, 21). Assessment of the above-mentioned parameters in dogs after a three-minute trotting test substantially improves the detection of clinically relevant signs compared to clinical examination at rest (6). The outcome of ET is directly associated with the overall respiratory function in a clinically relevant way. This test is currently routinely used in clinical cases to decide which dogs need upper airway surgery. Several kennel clubs such as the Kennel Club, UK (22) or Austrian Cynological Society OKV (23) recommend an ET assessment prior to breeding in some of the BOAS-affected breeds. Due to the subjective component, acceptance of ET results may however vary, particularly if they diverge from the owner’s impression of the dog’s respiratory function. Unfortunately, a dog’s exercise tolerance can be influenced by multiple factors such as ambient temperature, humidity and the dog’s stress level and overall fitness (24, 25). To obtain a quantitative assessment of respiratory function, a whole-body plethysmography (WBP) based assessment was developed for dogs (26). For WBP, dogs are placed in a barometric chamber and small pressure differences between inspiration and expiration are used to calculate respiratory function parameters such as inspiratory and expiratory peak flow and times, tidal volume and frequency. Liu et al. (17, 26) developed a statistical model that predicts a dog’s BOAS grade in the three-minute trotting test from WBP parameters. After validation of the model, WBP has been used as an independent diagnostic tool in follow-up studies [e.g. (6)]. While WBP is non-invasive, the technical equipment and time needed per measurement prevent its broader application (6). Also, the reproducibility of findings is unclear, since WBP for BOAS diagnostic has to date only been implemented in one clinic.

This study was designed to assess BOAS severity grade in a cohort of French Bulldogs using the currently accepted methods such as cranial anatomy measurements, ET as well as by WBP introduced recently. The second objective of the study was to explore the possibility of finding a reliable quantitative blood-borne marker of BOAS severity in French Bulldogs. The selection of blood parameters for the present study was based on the differences we have observed earlier when comparing blood samples of dogs of several brachycephalic breeds with those of age-and size-matching non-brachycephalic dogs (27). We suggested that the causes of these differences could originate in the intermittent hypoxic conditions associated with BOAS (2) and lower arterial oxygen pressure reported in brachycephalic dogs (28). As a response to the hypoxic challenge, NO production was reported in brachycephalic dogs (29). People with OSA (30, 31) and dogs with BOAS (27) are presented with upregulation in blood-borne oxidative stress markers. Those include an increase in plasma inflammatory cytokines in OSA patients and dogs with BOAS (29, 32, 33) and depletion of intraerythrocytic reduced glutathione stores (27). Signs of mild stress erythropoiesis (higher reticulocyte counts and an increase in abundance of immature reticulocytes) were detected in brachycephalic dogs (27). Furthermore, lipid metabolites varied between the two groups. Finally, stress leucogram (neutrophil-to-lymphocyte ratio) and increased plasma cortisol levels were detected in brachycephalic dogs (27). Based on these reports, we have selected parameters associated with hypoxia, inflammation, and stress to be tested in blood samples of a cohort of French Bulldogs for which the BOAS severity grade was defined using classical anatomical and functional tests.

## 2 Materials and methods

Privately-owned French Bulldogs were recruited via the University Small Animal Hospital’s Facebook page and by contacting breeders. To participate in the study, dogs had to be at least 12 months of age and have intact upper airways (i.e., no previous upper airway surgery). Dogs with lameness, heart disease, respiratory disease other than BOAS, or dogs treated with nonsteroidals or corticosteroids were excluded. Ethical approval for the collection of samples and clinical data from dogs was granted by the Veterinary Office of the canton of Zurich (ZH161/19).

**Figure 1:**
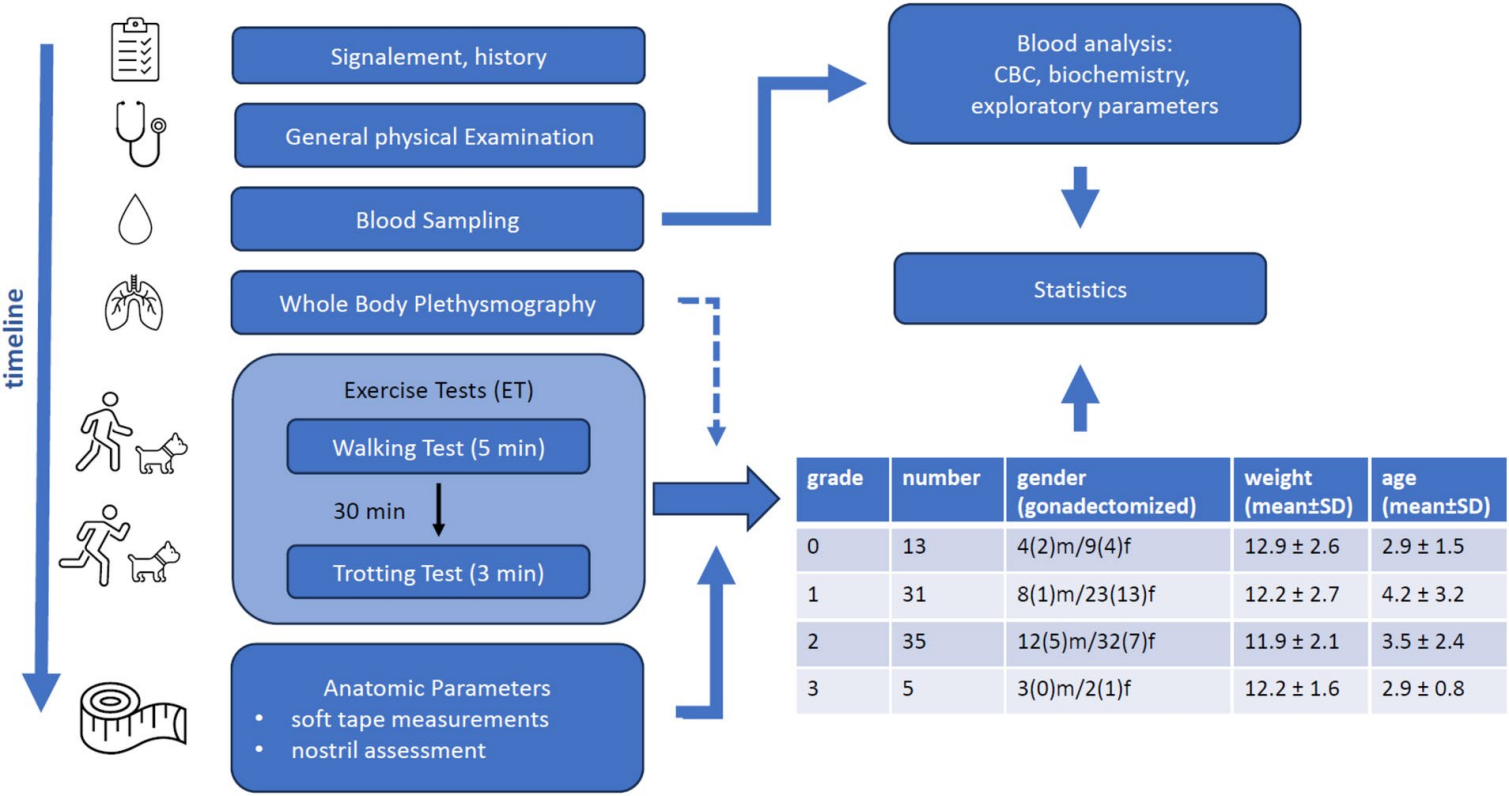
Overview of the study protocol including timeline of the procedures, statistical approaches and the resulting study cohort description stratified according to the BOAS severity grades.

Owners were given an outline of the study protocol and signed informed consent. An overview of the study protocol is shown in Figure 1. The dogs’ history was obtained with a focus on clinical signs of BOAS, and each animal underwent a general physical examination to ensure that the dog was (apart from potential clinical signs of BOAS) in a good general condition and fulfilled the inclusion criteria.

The clinical assessment of the dog continued with WBP, ET, and assessment of anatomical parameters. Clinical data was collected by three different veterinarians. Owners were allowed to stay with their dogs throughout the study.

### 2.1 WBP

Measurements were performed in one of two barometric chambers (model PLY-351 or PLY-361, EMMS Havant, Hampshire, UK). Initially, dogs below 15 kg bodyweight were placed in the smaller chamber (PLY-351). As dogs, however, commonly touched the walls of the chamber, which interfered with the measurements, we changed the cut-off to 7–8 kg following the protocols used by the group of Liu in Cambridge.

The obtained recordings of respiratory patterns were analyzed using the eDacq software (EMMS Havant, Hampshire, UK). The set-up was calibrated with 300 mL of air according to the manufacturer’s instructions before each session. During measurements, chambers were ventilated with 20 L/min room air. If humidity increased over 60–65% or CO_2_ over 0.4%, the doors were opened until humidity was below 50% and CO_2_ was at 0.04%. Baseline humidity was commonly around 50–60%. To avoid further increases once the dog was in the chamber, the outflow of an air conditioning device was directed at the openings from which fresh air was pumped into the chamber (distant from the chamber itself). Before the onset of recordings, all dogs underwent a training phase in which they were positively reinforced for entering the chamber and staying in the chamber for gradually increasing amounts of time. Once the dog was familiarized with the chamber, the outer door of the chamber was closed, and breath recordings were started. The owners were actively involved in acclimatizing their dogs to the chamber and stayed next to the box during the WBP measurement (i.e., within sight for the dogs). If a dog appeared stressed, it was released from the chamber, given a 5-min break, and then re-introduced to the chamber. Measurements were discontinued if a dog showed persistent stress-associated behavior such as panting, yawning, or scratching. The goal was to record a sequence of 20 consecutive breaths in the same position. Standing, sitting, or laying positions were all accepted, as long as the head was not rested on the floor. The recording had to be restarted if the position was changed. Head movements were tolerated if (a) the head returned to the original position within a few breaths (e.g., a quick look to the side) or (b) they were small enough to not change the overall alignment of the head, neck, and body. Average respiratory rates between 10 and 40 breaths per minute were eligible for analysis.

Candidate breath sequences were selected live during the recording. Definitive sequence selection was performed once data collection was completed to ensure consistent implementation of defined selection criteria. To assess the quality of the sequence, movement was graded as “invisible,” “minimal,” or “moderate” and an interruption index, defined as the number of not recognized or not selected breaths divided by 20, was calculated. If more than one sequence was obtained, the highest-quality sequence was selected for analysis.

From the 20 selected breaths, EMMS Data ACQuisition software calculated means (av) and standard deviations (sd) for various respiratory parameters, including respiratory rate (f, measured in breaths/min), time for inspiration (Ti, measured in s), time for expiration (Te, measured in s), peak inspiratory flow rate (Pif, measured in ml/s), peak expiratory flow rate (Pef, measured in ml/s), tidal volume (TV, measured in ml), and minute volume (MV = TV × f).

The statistical model developed by Liu et al. (17, 26) predicts BOAS grades based on the means and standard deviations of Te/Ti ratio (Te/Ti), Pef/Pif ratio (Pef/Pif), minute volume per body weight (MVBW, measured in ml/kg), and f. We were kindly provided access to a web application of this model, and further analysis of WBP data focused on those parameters (Te/Ti_av, Te/Ti_sd, Te/Ti_av, Te/Ti_sd, MVBW_av, MVBW_sd, f_av, f_sd).

### 2.2 ET

ETs followed the protocol performed by Riggs et al. (6) and were conducted in a corridor in the air-conditioned basement of the Small animal clinics with an ambient temperature of 20–21°C. A 5-min walking test was followed by a 3-min trotting test 30 min later. Owners were asked to walk or trot the dog on the lead at a speed of approximately 3–4 km/h in the walking test and 7–8 km/h in the trotting test. In cases of collapse, cyanosis, severe respiratory distress, or regurgitation, ETs were immediately stopped, and supportive care was initiated.

Immediately before and after both ETs, i.e., walking and trotting test, dogs were assessed for respiratory noises (including larynx auscultation), inspiratory effort, dyspnea, and cyanosis. BOAS grades 0–3 were assigned for each test separately according to Table 1. For further analysis, only the BOAS grades resulting from the respiratory functional grading before and after the trotting test were considered. Dogs with a history of collapse or cyanosis were not exercised and were directly assigned BOAS grade 3.

**Table 1:** Anatomical and functional indicators of BOAS severity grade [modified from Riggs et al. (6)].

BOAS grade	Pre-ET/post-ET	Abnormal respiratory noise^1^	Inspiratory effort^2^	Dyspnea^3^	Cyanosis	History of syncope or syncope during ET
0	Pre	Absent				No
	Post					
1	Pre	Not audible OR Mild stertor (and similar noises) Moderate intermittent stertor exclusively when sniffing Nasal whistle	Absent	Absent	Absent	No
	Post	Mild stertor (and similar noises) Moderate intermittent stertor solely when sniffing Nasal whistle	Absent OR mild	Absent	Absent	
2	Pre	Mild—moderate (all types of noises)	Mild—moderate	Absent	Absent	no
	Post	Moderate—severe (all types of noises) OR Mild stridor	Moderate—severe	Mild	Absent	
3	Pre	Moderate—severe (all types of noises)	Moderate—severe	Moderate—severe	If present or history of cyanosis => no ET	Yes => no ET
	Post	Severe	Severe	Severe	May be present; if noticed during ET => stop	During ET => stop

The dimension in which the dog showed most severe signs was relevant for the grading (e.g., a dog with mild stridor and mild inspiratory effort post-ET was assigned grade 2).

1 Abnormal respiratory noise according to Riggs et al. (6): mild = only audible at larynx-auscultation with stethoscope; moderate = intermittent noise audible without stethoscope; severe = constant noise audible without stethoscope. Stertor = lower-pitched snoring noise; stridor = higher-pitched noise. Mild stridor was classified as grade 2, whereas intermittent nasal stertor associated with sniffing was classified as grade 1, as described in Riggs et al. (6). In addition to stertor and stridor, we noticed nasal whistle, i.e., a moist nasal noise, in some dogs which was classified as grade 1 (personal communication J. Ladlow).

2 Inspiratory effort according to Riggs et al. (6): mild = “regular breathing patterns with minimal use of the diaphragm”; moderate = “evidence of use of the diaphragm and excessive thoracic wall movement with or without abdO_minal effort”; severe = “marked movement of diaphragm and excessive thoracic wall movement with or without abdO_minal effort”.

3 Dyspnea according to Riggs et al. (6): “Mild dyspnea, the dog is not relaxed when breathing; moderate dyspnea, irregular breathing, signs of discomfort (e.g., staring eyes, excessive sclera, grimace, ear taut backwards); severe dyspnea, irregular breathing with signs of breathing discomfort and difficulty in breathing”.

In addition to the parameters used for BOAS grading, rectal temperature was measured before and after each ET, and the temperature difference was calculated. Temperature measurement was omitted if a dog had shown signs of stress during previous temperature measurements (i.e., during the physical exam or before/after the walking test).

### 2.3 Anatomical conformation: nostril assessment and soft tape measurements

**Figure 2:**
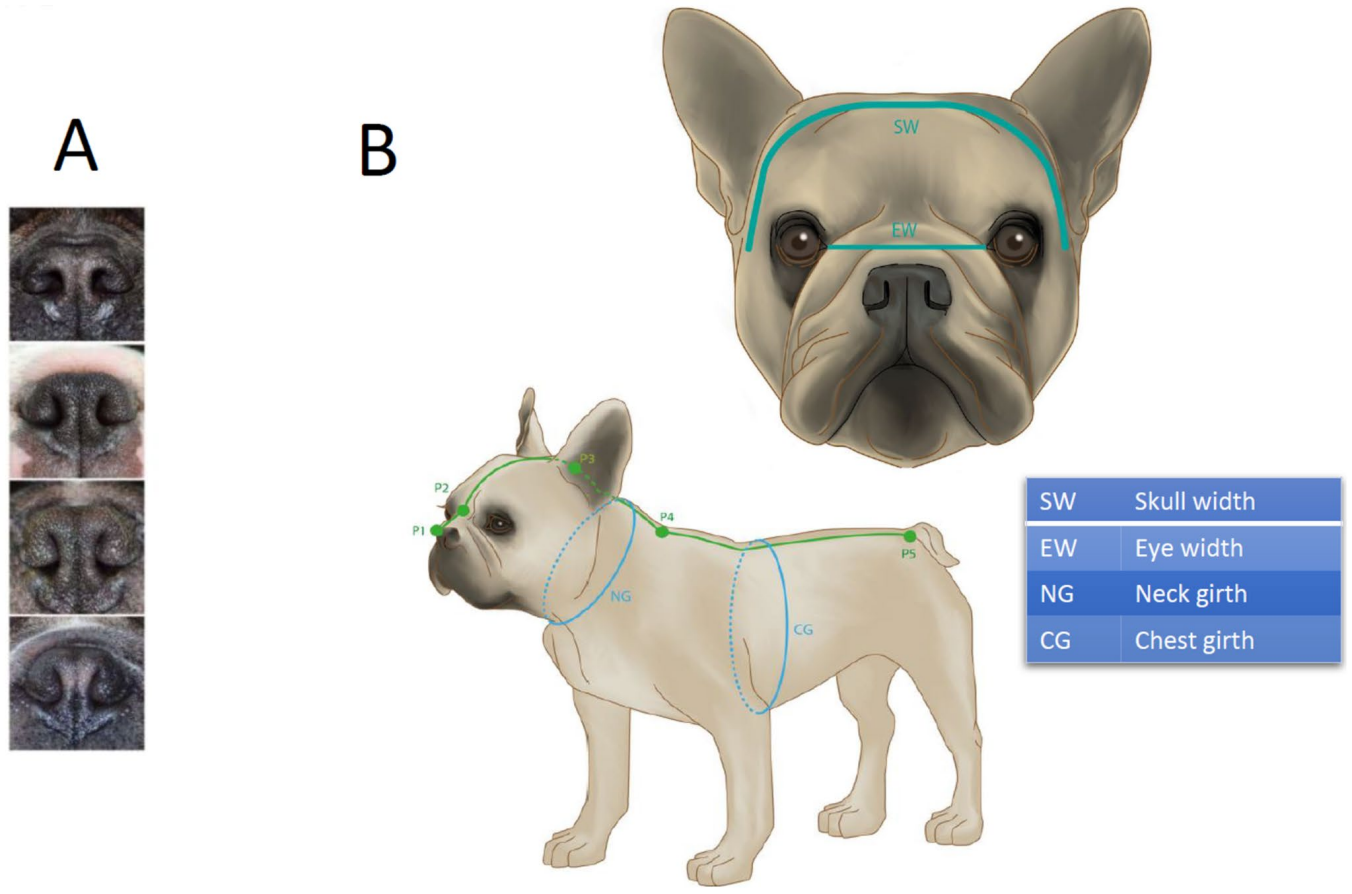
Stenosis grading illustrations **(A)** and soft tape measurements of head and body **(B)** as defined by Liu et al. (17): snout length = P1 to P2, from the rostral end of the nasal planum to the point between the left and right medial canthus; cranial length = P2 to P3, from the point between the left and right medial canthus to the external occipital protuberance; skull length = snout length + cranial length, i.e., P1 to P3; neck length, NL = P3 to P4, from to the external occipital protuberance to the point between the cranial angles of the scapulae; body length = P4 to P5, from the point between the cranial angles of the scapulae to the root of the tail; NG = neck girth, measured halfway between P3 and P4; CG = chest girth, measured directly behind the elbow where circumference is largest. **(B)** EW = eye width, measured as horizontal distance from medial canthus to medial canthus; SW = skull width, distance between left and right external zygomatic arch when the soft tape is placed on the head.

Assessment of anatomical conformation closely followed the procedures described in Liu et al. (17). Nostril conformation was assessed on a four-point scale, ranging from open to severely stenotic. Open nostrils were defined as nostrils without visible stenosis. Mildly stenotic nostrils were characterized by some degree of narrowing, which still allows for dorsolateral movement of the nostril wings upon exercise. In contrast, the nostril wings of moderately stenotic nostrils do not move after exercise, and the dorsal part of the nostril is (almost) closed (i.e., the lateral and medial walls are touching). In severely stenotic nostrils, both the dorsal and the ventral aspects of the nostril are closed, resulting in mouth breathing when walking or even at rest. An example for each grade is given in Figure 2A. For further analysis, moderately and severely stenotic nostrils were considered clinically significant stenosis (NS+), whereas open and mildly stenotic nostrils were summarized as clinically not significant stenosis (NS−).

Soft tape measurements of the head, neck, and body were taken as shown in Figure 2B to calculate the following ratios as defined in Liu et al. (17):

- Craniofacial ratio (CFR): snout length/(snout length + cranial length)
- Eye-width ratio (EWR): eye width/skull width
- Skull index (SI): skull width/skull length
- Neck girth ratio (NGR): neck girth/chest girth
- Neck length ratio (NLR): neck length/body length

### 2.4 Blood parameters

A total of 6 mL of venous blood was collected from the jugular vein, the vena cephalica or vena saphena. All methods used had been described previously (27). Clinical hematology included the complete blood count (CBC), reticulocyte count and maturity (low, medium, and high fluorescence), reticulocyte hemoglobin, and thrombocrit as well as total protein, albumin, bilirubin, glucose, urea, creatinine, cholesterol, triglycerides, alkaline phosphatase, alanine aminotransferase, creatine kinase, lipase, lactate dehydrogenase, phosphate, Na^+^, K^+^, Cl^−^ total Ca^2+^ and the ionized Ca^2+^. Furthermore C-reactive protein (CRP) was assessed as a clinical marker of inflammation.

Redox state markers (bulk thiols and non-protein reduced thiols) were assessed using monoromobimane (Mbbr) fluorescence and Ellman’s regagent, respectively, as described elsewhere (27). Osmoscane mode of Laser rotational red cell analyzer (Lorrca) was used to assess deformability, RBC hydration and membrane osmotic stability as described by van Cromvoirt et al. (34). Membrane surface area was estimated using eosim 5-maleimide (EMA) fluorescence (27). Avoximeter 4,000 was used to assess oxy-(O_2_Hb), carboxy-(COHb) and met-hemoglobin (metHb). CO-oximetry was performed on the freshly drawn samples (within 15 min after blood collection). Precautions were taken to avoid reoxygenation.

### 2.5 Statistical analysis

#### 2.5.1 Analysis of demographic data, ET, and anatomical parameters

All analyses described in this section were performed in R version 4.2.1 (35), and significance levels are set at 5% unless stated otherwise. Descriptive statistics were generated for demographic data, respiratory functional grading in the walking and trotting tests and temperature differences across walking and trotting test, as well as for nostril conformations. Missing values were amended with the R-package missForest (36).

Possible associations of temperature differences in the walking and trotting test with BOAS severity grade were tested using one-way Analysis of Variance (ANOVA). A proportional-odds ordered logistic regression [package MASS, (37)] was used to test for the impact of nostril grade (reference category 1), anatomical ratios obtained from soft tape measurements (centered at respective means), and temperature difference in the trotting test on the odds of a dog being assigned to a lower BOAS grade. Age, gender (reference category female), neuter status (reference category intact), body condition score (reference category 5) and weight (centered at the mean) were included as control variables. Non-relevant collinearity between predictors was defined as an adjusted generalized variance inflation factor below 1.6 and checked with the car package (38).

#### 2.5.2 Analysis of WBP data

Initially, WBP data was analyzed with the Cambridge web application. This application gives an overall BOAS score between 0 and 100 and depicts a radar plot with the likelihood of each BOAS grade (0–3). Radar plots with likelihoods greater than 0% in more than 2 grades or in non-neighboring grades are considered implausible (Liu, personal communication). Subsequently, we performed a quadratic discriminant analysis (QDA), according to Liu et al. (17, 26) with our data. BOAS status, i.e., BOAS +/− resulting from the trotting test, was used as reference. Te/Ti_av, Te/Ti_sd, Te/Ti_av, Te/Ti_sd, MVBW_av, MVBW_sd, f_av and f_sd of our study dogs were used to build a model classifying dogs as BOAS+ or BOAS−. Due to our smaller sample size, the classification of dogs was limited to the binary BOAS+/− instead of 4 grades used by Liu et al. (17, 26). The statistical approaches and the respiratory parameters used for BOAS grading in this study were identical to those reported by Liu and colleagues.

#### 2.5.3 Analysis of blood parameters

One-way ANOVA (on ranks or on raw data depending on the outcome of normality test), when not stated otherwise, was used to identify statistical significance of differences in the individual blood parameters between the dogs with BOAS grades 0–3. These parameters included oxyhemoglobin (O_2_Hb), carboxyhemoglobin (COHb), Fluorescence intensity of Mbbr in reticulocytes, reduced glutathione GSH, Reticulocyte count, Reticulocyte Hb, Mean fluorescence reticulocytes, O_min, O_hyper, monocyte count and Mean platelet volume (MPV). Bonferroni-Holm correction was applied for multiple testing.

A linear regression was used to assess the possible associations between the COHb and O_2_Hb, as well as between Reticulocyte count and reduced thiol abundance measured as Mbbr fluorescence intensity in reticulocytes/high fluorescence intensity cells.

To better understand the correlations and dependencies between the variables, a principal component analysis (PCA) was used. A PCA represents a multidimensional dataset with typically two principal components which are linear combinations of the original variables. (39). PCA was performed using R software version 4.2.1. Parameters were scaled before entering into the analysis.

Additionally, to select the most representative variables and to rank them with respect to BOAS status, the functions available in the *varrank* (40) and the *caret* (41) packages were used. The *varrank()* function was used with the Estevez score function model and the Sturges approach for discretization. The resulting scores for each variable are plotted together with an information about relevancy and redundancy. The *caret* package allows for a model-free variable selection using a recursive partitioning approach.

## 3 Results

### 3.1 Clinical results

Of the 102 recruited dogs, 84 completed the study and were included in the analysis (Figure 1). Reasons for exclusion were uncooperative behavior (11 dogs), not fulfilling inclusion criteria (5 dogs), and clotting of blood samples (2 dogs). Two-thirds of the included dogs were female (57 dogs), and roughly 40% (33 dogs) were gonadectomized. Their age ranged from 1 to 13 years (median 2 years 8 months). Further information on the dogs participating in the study may be found on in Supplementary Table 1 and on Zenodo platform (DOI:10.5281/zenodo.13358272).

#### 3.1.1 ETs

No dog collapsed or developed severe respiratory distress during the ETs. One trotting test was terminated 30 s earlier due to suspected cyanosis, and two dogs were directly classified as grade 3 due to a history of previous collapse.

Approximately half of the participants had BOAS grades 0 and 1. Among the remaining 40 dogs, only 5 were severely affected and classified as BOAS grade 3 (Figure 1 and Supplementary Table 1). None of the following dog characteristics (gender, reproductive status, age, weight) was associated with BOAS severity grade.

Overall, BOAS grades (0–3) had a good agreement between the walking and trotting test; 73% of the dogs who were exercised (60/82) were assigned the same grade in both ETs. In those dogs that scored differently in the walking versus trotting test, the grade assigned in the trotting test was typically higher than the grade assigned in the walking test (18 dogs). Although a minority of dogs scored higher in the walking test (4 dogs), the final classification was based on the trotting test only.

Temperature data were obtained for 75 walking and 74 trotting tests and missing data was imputed (Supplementary Table 1). Changes in body temperature during ETs were typically small, with a median of 0.18°C (range: −0.6 to 0.8°C) in the walking and 0.2°C (range: −0.6 to 1.1°C) in the trotting test (Supplementary Table 1). Due to the small number of grade 3 dogs and the high proportion of imputed values in this category, grade 3 was excluded from the ANOVA. The results of the ANOVA, including grade 3 dogs, are provided in the supplements. Changes in body temperature did not significantly differ between BOAS grades in either test (walking test, BOAS grade in walking test: *F* = 0.01, *p* = 0.976; trotting test, BOAS grade in trotting test: *F* = 2.734, *p* = 0.102). It is noteworthy that increases in rectal temperature of more than 0.4°C in the trotting test were exclusively found in BOAS+ dogs (grades 2–3). Temperature difference in the trotting test was, however, not a significant predictor of the odds of having a lower BOAS grade in the ordered logistic regression (Table 2).

**Table 2:** Results of the ordered logistic regression.

	B (SE)	OR	95% CI	*p*-value	
Nostril grade 2	1.04 (0.99)	2.83	(0.41, 19.56)	0.29	
Nostril grade 3	2.75 (1.18)	15.58	(1.55, 156.59)	0.022	*
Nostril grade 4	3.40 (1.28)	30.01	(2.422, 372.02)	0.01	*
CFR	−2.75 (6.09)	0.06	(0.97, 6.67)	0.65	
EWR	26.20 (11.51)	2.40 e+11	(38.357, 1.506 e+21)	0.025	*
SI	2.14 (0.93)	8.46	(1.38, 51.94)	0.024	*
NGR	−1.64 (4.70)	0.19	(0.194, 5.74)	0.73	
NLR	−12.95 (4.62)	0	(0, 0.02)	0.006	*
Sex male	0.91 (0.70)	2.48	(0.63, 9.80)	0.20	
Neuter status yes	−1.09 (0.54)	0.34	(0.12, 0.97)	0.047	*
BCS 3	0.58 (1.218)	1.78	(0.17, 19.08)	0.64	
BCS 4	0.90 (0.57)	2.47	(0.8, 7.61)	0.12	
BCS 6	0.19 (1.10)	1.21	(0.14, 10.54)	0.86	
BCS 7	−0.66 (1.91)	0.51	(0.012, 21.86)	0.73	
Weight	−0.17 (0.13)	0.85	(0.65, 1.10)	0.21	
Age (months)	−0.007 (0.007)	0.99	(0.98, 1.01)	0.31	
Temperature difference ET (°C)	0.58 (1.14)	1.78	(0.19, 16.58)	0.61	

B = regression coefficient (on log odds scale); SE = standard error; OR = odds ratio; CI = confidence interval; *p < 0.05. The reference is a 12 months old female intact dog of average weight, with grade 1 nostrils, average CFR, EWR, SI, NGR and NLR, and 0 temperature difference in the ET. Coefficients with positive signs reduce the odds of being in a lower BOAS grade, i.e., increase the probability of having a higher BOAS grade, whereas coefficients with negative signs increase the odds of being in a lower BOAS grade, i.e., reduce the probability of having a higher BOAS grade.

#### 3.1.2 WBP

**Figure 3:**
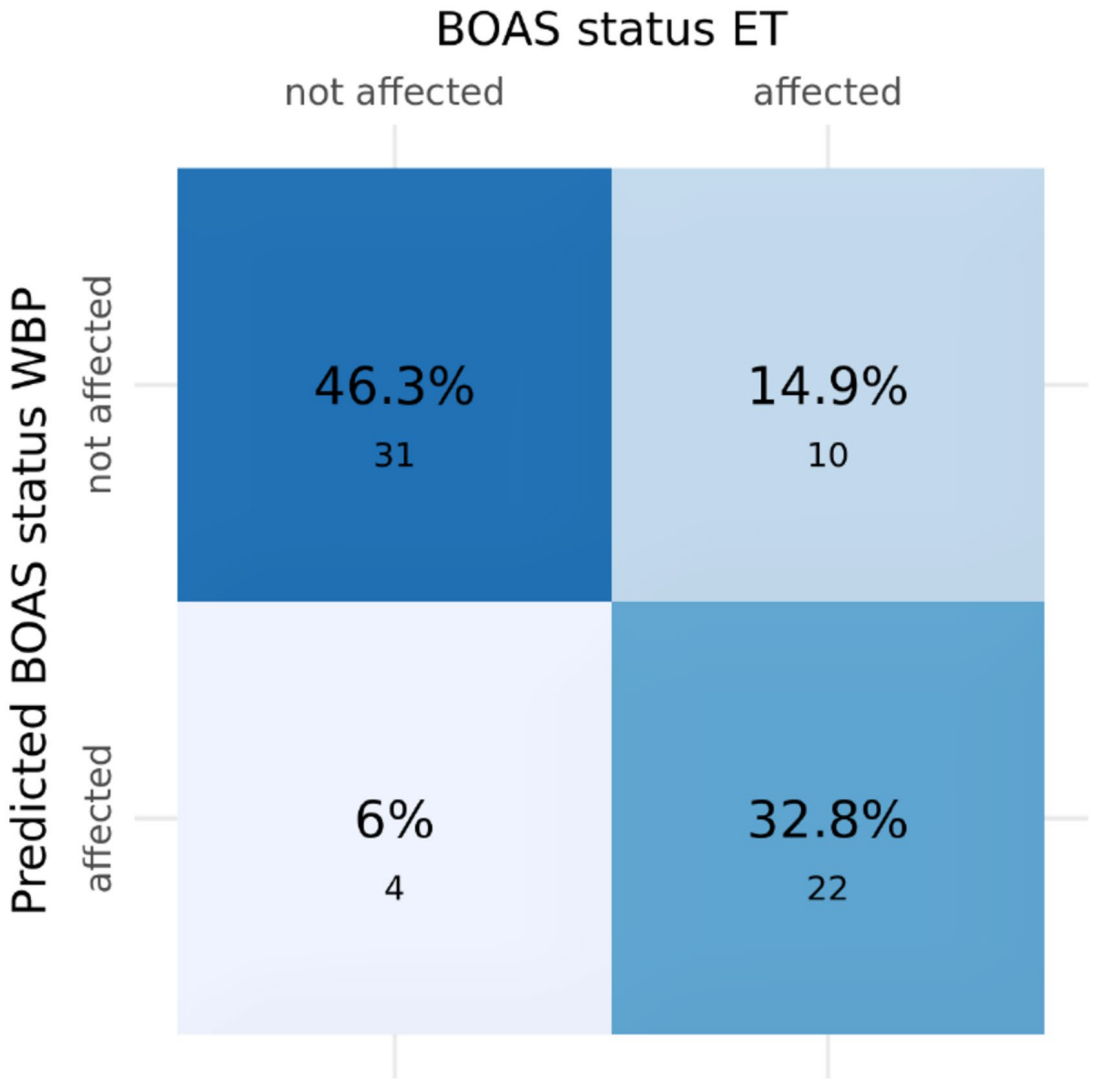
Confusion matrix of BOAS status in the trotting test versus predicted BOAS status based on WBP. Numbers in the quartiles are the number of dogs in each category. Sensitivity: 0.688, Specificity: 0.886, Positive Predictive Value: 0.846, Negative Predictive Value: 0.756, Accuracy: 0.791.

Sufficient quality data, i.e., a sequence of 20 breaths fulfilling the defined criteria, was available in 67 dogs. WBP data analyzed with the Cambridge web application often produced implausible radar plots. Only in 18 dogs, the BOAS index calculated by the Cambridge Web application coincide with the BOAS grading resulting from ET. To assess the power of the obtained data in predicting BOAS severity grade by means of WBP, we performed QDA on the available data using BOAS grading based on ET as the true classification. This resulted in a model with 79.1% accuracy (Figure 3). As the classification of each dog’s BOAS grade as well as the BOAS index relied on respiratory functional grading, we could not use the BOAS index in further statistical analyses for associations between blood parameters and clinical signs. Further analysis of associations between any of the anatomical or blood parameters and the BOAS grade was based on the respiratory functional grading assigned in the ET trotting test only.

#### 3.1.3 Nostril assessment and soft tape measurements

**Figure 4:**
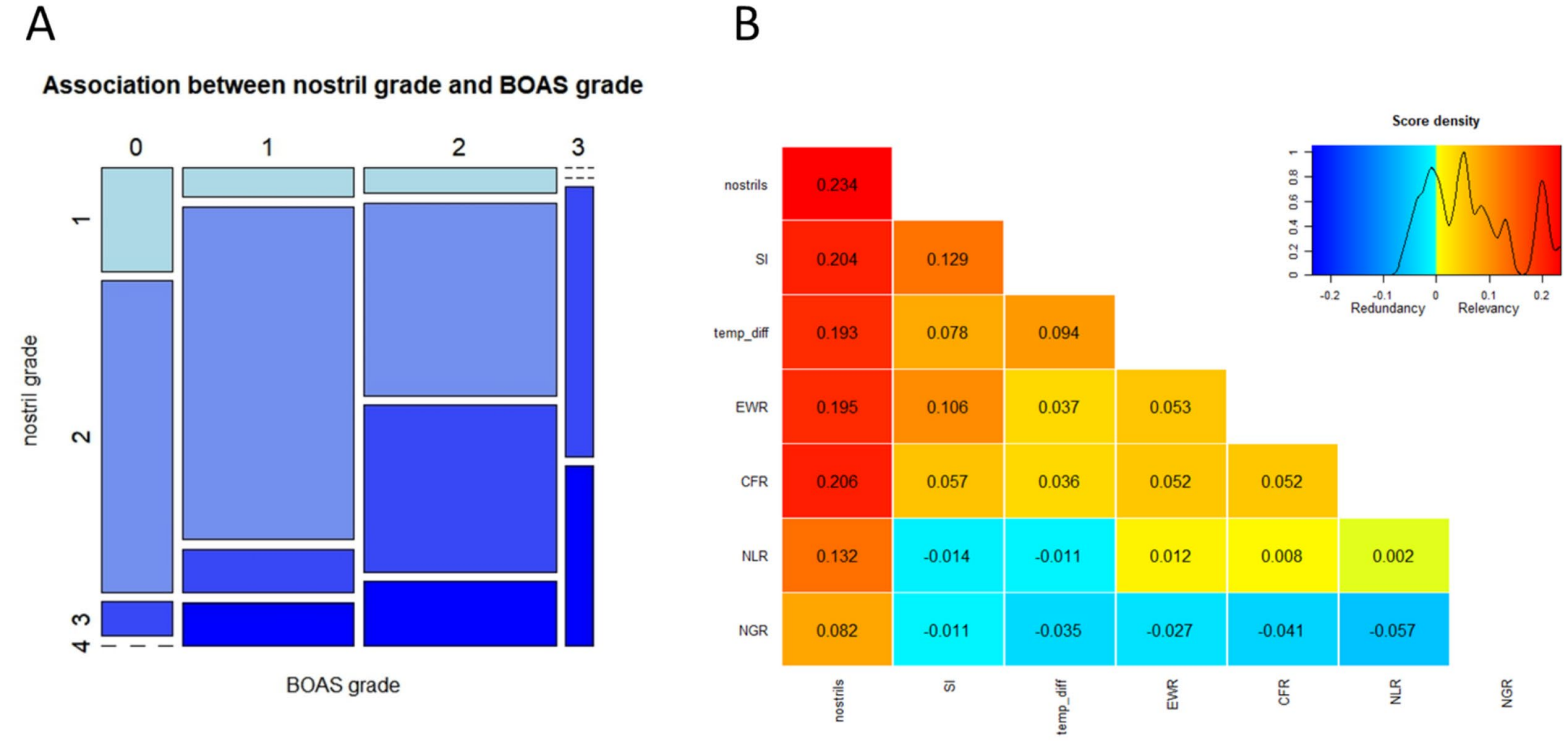
Association between nostril grade and BOAS grade. **(A)** Rectangles in the mosaic plot are proportional in size to the number of dogs falling in each category. No dog with BOAS grade 0 had severely stenotic nostrils (grade 4), and no dog with BOAS grade 3 had open nostrils (grade 1) in our study cohort. **(B)** Ranking of the anatomical parameters according to the power of association with BOAS severity grade using the *varrank* algorithm. The heatmap and the numbers reflect the relevance scores. Stenosis of nostrils shows the highest degree of association with BOAS severity grade based on the functional test. Also relevant are the ratio between skull width and skull length (SI), temperature increase after 3 min trotting test (temp_diff), the ratio between eye width and skull width (EWR) and the ratio between snout length and skull length (CRF). The ratio between neck length and body length (NLR) and the ratio between neck girth and chest girth (NGR) were scored as less relevant predictors of BOAS severity.

Anatomical conformation data of one dog was not saved correctly due to technical issues. Open or mildly stenotic nostrils were found in 7 and 47 dogs, respectively. Stenotic nostrils were present in 30 dogs, i.e., more than one-third of the animals. Figure 4A illustrates the distribution of nostril grades among dogs of different BOAS grades. The ordered logistic regression revealed that, compared to dogs with open nostrils, dogs with stenotic nostrils were significantly more likely to have a higher BOAS grade (Table 2).

Soft tape measurements of the head were performed in 79 dogs only, since five dogs did not tolerate them. The missing values were imputed. Higher EWR, higher SI and lower NLR were significantly associated with a higher probability of having a higher BOAS grade in the ordered logistic regression. Dogs with stenotic nostrils, eyes wide apart, a broad skull and short neck are thus more likely to have a higher BOAS grade (Figure 4A). *Varrank* function was used to rank the anatomical parameters with regard to their association with BOAS grades (Figure 4B). This approach supported the importance of nostril grading in predicting the BOAS severity. Nevertheless, there is considerable overlap between BOAS grades in all ratios, as shown in Table 2. Among the control variables, age, sex, weight and BCS were not significantly associated with higher probabilities of a higher BOAS grade, but intact dogs were more likely to have a higher BOAS grade in our study cohort.

### 3.2 Blood parameters

#### 3.2.1 RBC turnover and hypoxic markers

**Figure 5:**
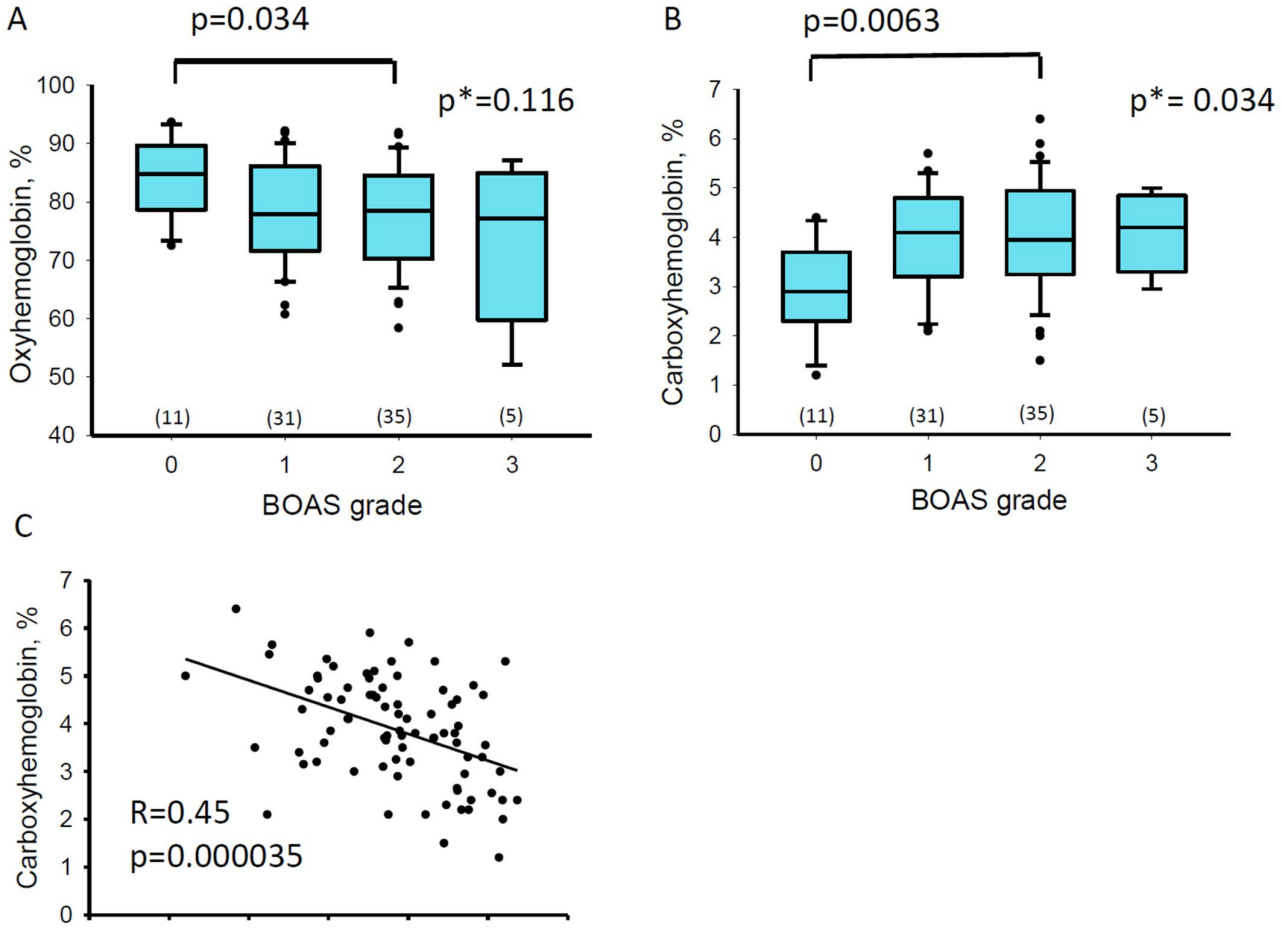
Co-oximetry results. Box blots show the Oxyhemoglobin, O_2_Hb **(A)** and Carboxyhemoglobin, COHb **(B)** as a function of BOAS grade. Numbers in brackets are the number of dogs in each group. One-way ANOVA test was applied for all the BOAS grades as well as the Student’s test to test for the differences between BOAS groups 0 and 2. Numbers are *p*-for the Students test and p* for the *p*-value of the ANOVA tests. **(C)** Correlation between the O_2_Hb and COHb levels for all the study participants. *R* and *p* values are for the linear regression analysis.

Reduction in hemoglobin oxygen saturation (SO_2_) with aggravating clinical signs of BOAS did not reach the statistical significance threshold (One-Way ANOVA *F*-test). However, a comparison of SO_2_ between the BOAS grade 0 and grade 2 groups reached significance (Student’s t-test, Figure 5A). The increase in severity of BOAS was associated with an increment in COHb levels in blood of the study participants, as proven by the one-way ANOVA test (Figure 5B). The content of COHb was significantly higher in the BOAS grade 2 group compared to the grade 0 control (Figure 5B). A strong negative correlation was observed between the hemoglobin oxygen saturation and the carboxyhemoglobin levels (Figure 5C).

A trend toward an increase in reticulocyte counts (Supplementary Figure S1A), as well as a shift to a more immature phenotype of these cells (as follows from the higher abundance of RNA remnants, Supplementary Figure S1B), was observed as the BOAS severity progressed from grade 1 to grade 3. Furthermore, reticulocytes from dogs with BOAS grades 2–3 were presented with more oxidative stress. Reticulocytes contain substantially more reduced thiols that can be visualized by fluorescence intensity (Supplementary Figure S2A). Redox state of reticulocytes, and their abundance could be reflected by the fluorescence intensity (Supplementary Figure S2A,B) and reticulocyte counts (Supplementary Figure S2C) in the so-called “high Mbbr fluorescence cell fraction” (exemplified in green, Gate B, in the inset of Supplementary Figure S2A). Fluorescence intensity of “high Mbbr fluorescence cell fraction” decreased in the blood from dogs with BOAS grades 2 and 3 (Supplementary Figure S2D). Furthermore, a decrease in the intraerythrocytic GSH content was observed with an aggravation of BOAS severity from grade 0 to grade 2, reaching *p* = 0.054 (Supplementary Figure S2E). However, GSH depletion was not observed in RBCs from dogs at the highest severity grade 3 (Supplementary Figure S2E).

No statistically significant differences were observed in RBC deformability between the dogs with BOAS severity grades 0–3, but a trend toward better tolerance of hypoosmotic conditions and a lower tolerance threshold to hyperosmotic conditions suggested some degree of dehydration of RBCs (Supplementary Figure S3A,B). Finally, the other blood cells showing the BOAS grade-specific differences included mean plate volume (Supplementary Figure S3C) and monocyte counts (Supplementary Figure S3D).

#### 3.2.2 Principal component analysis (PCA) and other statistical models

Principal component analysis was performed for the following variables: COHb, O_2_Hb, intraerythrocytic GSH, fluorescent intensity of high fluorescence Mbbr RBC fraction, O_min, O_hyper, monocyte count and platelet volume. The inclusion of reticulocyte counts into the PCA as a variable explained over 98% of the variance in the data by this variable (data not shown). Therefore, reticulocyte count and maturation state were omitted in the PCA but used when the importance of various parameters for prediction of the BOAS severity grade was tested using *varrank* and *caret* algorithms.

Presented in Supplementary Figure S4A is a score plot where the loadings of the chosen variables into the principal components 1 and 2 explain 21.22 and 17.03 percents of variance, respectively. The score plot reveals the opposing trends for O_2_Hb and COHb. The COHb, along with O_min and O_hyper contributed mainly to the first principal component. The redox markers GSH and Mbbr fluorescence intensity of reticulocytes as well as MPV were the major contributors of the second component. The O_2_-Hb variable is equally presented in both principal components.

Principal components 1 and 2 were tested for their association with the BOAS severity grade. The ANOVA test revealed a significant association between the component 1 (Supplementary Figure S4B), but not principal component 2, and severity of the disease.

To further reveal the importance of the selected variables for the prediction of BOAS severity *varrank* and *caret* algorithms were used. The variables of interest included, along with those used for the PCA, the blood parameters associated with reticulocytes [reticulocyte counts, the fraction of medium fluorescence reticulocytes (MFR)], and reticulocyte hemoglobin content. The *varrank* algorithm revealed reticulocyte count and the reticulocytes’ proprieties as the most relevant predictors of the severity grade, along with COHb and intraerythrocytic GSH content (Supplementary Figure S4C). The alternative *caret* model gave the highest importance score to the oxyhemoglobin followed by carboxyhemoglobin, reticulocyte Hb and reticulocyte count, as well as fluorescence intensity of the young cells and, finally, O_min (Supplementary Figure S4D).

## 4 Discussion

Increasing the quality of life and avoiding suffering and premature death requires selective breeding toward a robust BOAS-free phenotype of brachycephalic dog breeds (42). To achieve this goal reliable identification of BOAS severity grades is crucial. Choosing between the most invasive but more precise imaging techniques requiring anesthesia and less invasive and less precise morphometric and functional tests, the owners and the vets prefer the latter. We have compared several morphometric and functional approaches. Based on our findings, ET gives the most reliable and satisfactory results in BOAS-grade detection for this brachycephalic breed.

### 4.1 Clinical grading approaches

The trotting test and the assessment of nostril width used in this study correspond to the Cambridge Respiratory Function Grading Scheme (RFGS). The implementation of this scheme for English Bulldogs, French Bulldogs and Pugs has meanwhile been recommended by the Fédération Cynologique Internationale (FCI) to all affiliated national breeding associations. The RFGS protocol focuses on the detection of respiratory distress and overall exercise tolerance; however, it does not explicitly assess for hyperthermia. In English Bulldogs the severity of BOAS increased the temperature change after submaximal exercise tests (21). Hence, we included rectal temperature measurements immediately before and after the exercise. Although a temperature increase of 0.5°C or more after performing the trotting test was only found in BOAS+ dogs, we did not detect significant differences in rectal temperature changes between BOAS+ and BOAS− French Bulldogs, which is similar to earlier findings in French Bulldogs (43, 44). However, in all three studies, the number of severely affected French Bulldogs was low, e.g., in our study only one of the five dogs graded BOAS 3 had temperature measurements before and after ET’s. Even if the determination of the temperature difference does not allow BOAS grading in the French Bulldog, the detection of a temperature increase of 0.5°C or more in the trotting test supports the diagnosis of BOAS + (45).

In addition to the ETs, we used the WBP as a recognized benchmark method (44) to determine the suitability of the selected blood parameters as future screening parameters. In our study, however, the agreement between BOAS grades predicted by the Cambridge web application and ET was poor (Figure 3). There are several possible explanations: first, the validation sample mainly consisted of show dogs (26), whereas our sample included primarily pet dogs not trained to sit calmly in a box. This could affect the quality of raw WBP data and thus the classification accuracy. Second, the selection of suitable breath sequences for analysis introduced a subjective component, so that highly standardized procedures could be required for reproducible findings. Third, we were not successful when using the web application developed by Cambridge at a different location (in Zurich), even when using an identical plethysmograph with the settings specified by Cambridge. Retraining the classification model using the list of parameters proposed by Liu et al. (26) but based on our WBP dataset resulted in an accuracy of 80%. While those parameters are physiologically plausible, we believe that higher classification accuracy could be achieved using different combinations of parameters for our specific study cohort and experimental setting. Due to the comparatively small sample size, we did not explore alternative parameter combinations. Even if the classification accuracy could have been improved, the question remains how much WBP could improve BOAS grading. In the meantime, trotting tests were shown to achieve a high sensitivity in severity grading of BOAS (44).

Craniofacial anatomic measurements are/were used for breeding restrictions to prevent the exaggeration of fronto-facial “baby-like” features such as relatively larger foreheads and eyes, which are related with BOAS and other health concerns.

However, in line with other studies [e.g., (17), our findings (Table 2)] do not support CFR as a robust predictor of higher BOAS. While three ratios derived from soft tape measurements were significantly associated with a higher probability of higher BOAS grade in our study, the identified ratios EWR, SI and NLR overlap only partially with those found by Liu et al. (17), NLR and NGR. Due to the considerable overlap of values between BOAS grades and the mediocre interrater reliability of soft tape measurements (17), soft tape measurements on their own lack robust predictive power sufficient for reliable BOAS grading. Stenotic nostrils on the other hand are clearly associated with a higher risk of BOAS, as shown by our data (Table 2) and in a previous study (17). Corrective surgery such as rhinoplasty may significantly improve the outcome of ETs, but not of the health status of the offspring of BOAS-prone parents. Hence, reporting of C-sections and surgeries is introduced by the British Kennel Club to exclude dogs with naturally severe BOAS from the breeding program (46). As stenotic nostrils are just one of several anatomical abnormalities contributing to BOAS, this approach may help to prevent the consequences of too narrow breeding selection. Particularly at the intermediate levels of the respective four-level grading systems, dogs may have mildly stenotic nares, but present as BOAS grade 2 in the trotting test, or vice versa may not be classified as clinically BOAS-affected despite moderately stenotic nostrils. Interestingly, neutered dogs in our study were less likely to have a higher BOAS grade (Table 2). This finding is not in line with the previous report on the lack of effect of neuter status in French Bulldogs and Pugs on BOAS severity, and a higher probability of neutered Bulldogs being affected by BOAS (17). The risk of anesthesia and the fear of neutering causing a worsening of clinical signs might explain, why dogs with more severe signs are less likely to be gonadectomized.

To date, studies linking anatomical conformation and BOAS grade in an ET are, to the best of our knowledge, cross-sectional. Longitudinal investigations might identify anatomical risk factors for clinical sign progression in dogs of BOAS grade 1 or 2 at the time point of screening. Prospective studies of Aromaa et al. elucidating the development of clinical signs of BOAS with time could also be helpful in better understanding the dynamics of the BOAS pathology (47). One more approach that could be undertaken to reduce the extreme anatomical manifestations resulting in BOAS is genetic screening for polymorphisms associated with severe phenotypes. Unfortunately, the studies suggest the involvement of multiple genes and deleterious alleles in the characteristic brachycephalic anatomy causing BOAS in different breeds making the selection of “healthy alleles” complicated (48). However, some of the polymorphisms, such as the c.2786G>A missense variant of the gene coding for the ADAM metallopeptidase with thrombospondin type 1 motif 3 (*ADAMTS3*) seem to be one of the promising candidates for a “genetic marker” of predisposition to BOAS development (49).

### 4.2 Impact of BOAS on red blood cells of French Bulldogs

Dogs with higher BOAS grades are likely to experience more frequent episodes of hypoxia, associated by sleep apnea or increased physical activity (2). While chronic hypoxia increases red blood cell volume and hemoglobin concentration to improve tissue oxygen delivery, neither an increase of blood hemoglobin concentration nor erythrocytosis was associated with BOAS severity in our study population. This finding is similar to that in OSA patients, where only in 9 out of 527 patients had polycythemia (50). In our previous study, in which blood parameters of brachycephalic dogs were compared with those in non-brachycephalic dogs, we observed the signs of stress erythropoiesis such as an increase in reticulocytes counts and an increase in the abundance of immature reticulocytes (27). In the present study, the differences in these parameters did not reach significance when the dogs with BOAS grade 0–3 were compared with each other (Supplementary Figure S1). However, reticulocyte count and reticulocyte hemoglobin and redox state were predicted to be strongly associated with BOAS grading by *varrank* and *caret* models (Supplementary Figures S4A, B). Alterations in RBC turnover associated with the severity hypoxic condition can also be suggested from an association between the oxy-and carboxy-hemoglobin levels (Figure 5). Carboxyhemoglobin appeared to be the blood parameter showing the strongest association with BOAS severity and was clearly upregulated with an increase in severity grade from 0 to 2 (Figure 2B). Carboxyhemoglobin is accepted as a robust clinically accepted predictor of hemolytic activity (51). Hemolysis was reported to counterbalance the augmented erythropoiesis in the OSA patients (31). In human patients with OSA an increase in plasma bilirubin concentrations were detected in the morning compared to the pre-sleep levels (52) suggesting that apnea may be associated with hemolytic episodes.

One more feature of RBCs of brachycephalic dogs includes oxidative stress, manifested as GSH depletion (27). A modest reduction in the intraerythrocytic GSH levels could also be seen as BOAS severity progressed from 0 to 2 (Supplementary Figure S2E). Unfortunately, deoxygenation of hemoglobin to 50% or more may result in the release of extra GSH molecules stored within oxygenated hemoglobin resulting in an increase in free GSH in the cells, which is not associated with amelioration of oxidative stress (53). This behavior makes GSH a poor prognostic marker of intermittent hypoxia. One more interesting finding of the present study is trend to oxidation of protein thiols in reticulocytes of BOAS grade 2 (and probably grade 3) dogs (Supplementary Figure S2). Whether this trend is associated with the stress erythropoiesis condition or with the systemic oxidative stress as discussed earlier (27), remains to be explored.

### 4.3 Limitations and the outlook of the study

We limited our research to one breed, the French Bulldog, to keep variances as small as possible. A statement is therefore only possible for this breed. It cannot be ruled out that French Bulldogs, which differ significantly from other dog breeds in the count of disorder predispositions (42) may also show differences in their blood parameters. However, since several of their “ultra-predispositions” were associated with their brachycephalic conformation, it might be assumed that the blood parameters associated with BOAS severity may be shared by other brachycephalic breeds, not only by French Bulldogs. The small number of animals in the “grade 3” group makes the data unbalanced and conclusions on the changes in blood parameters in brachycephalic dogs severely affected by BOAS questionable.

As follows from our study, none of the single-standing blood parameters qualified as a self-standing predictor of BOAS severity at the stages of 1–2 grades. Those requiring attention are CO-hemoglobin, reticulocyte counts, and their maturation, as well as RBC redox markers. They may be assessed as individual parameters or used to produce a compound variable like the principal component 1 in our PCA analysis (Supplementary Figures S4A, B). In our present study, we failed to reliably measure plasma nitric oxide derivatives NO_2_^−^/NO_3_^−^ as plasma samples often showed signs of hemolysis, which may interfere with the measurements. Thus, we cannot exclude that NO levels may add to the potential markers of BOAS severity, as higher levels of nitrite and nitrate in plasma was found in BOAS dogs earlier on (27, 29).

Much more research is needed to determine whether blood values are ultimately suitable as predictors of BOAS severity, which is a prerequisite to targeted selective breeding for healthier phenotype in brachycephalic breeds. According to our study results in French Bulldogs, promising candidates could be CO-hemoglobin, reticulocyte counts, and their maturation, as well as RBC redox markers. To avoid the animal welfare-related consequences of extreme brachycephalic breeding, we recommend for the time being, that all dogs of brachycephalic breeds undergo ET by trained veterinarians analogous to the Respiratory Function Grading Scheme of the Kennel Club and University of Cambridge’s, before both showing and breeding. Since only a small proportion of dogs are bred under Fédération Cynologique Internationale rules, it is crucial for the welfare of brachycephalic dogs bred in the future, that the screening test is required by law for all brachycephalic breeding dogs.

## Data Availability

The datasets presented in this study can be found in online repositories. The names of the repository/repositories and accession number(s) can be found in the article/Supplementary material.
