# Supplementary material for: Anatomical, functional, and blood-born predictors of severity of brachycephalic obstructive airway syndrome severity in French Bulldogs
Source: Front Vet Sci. 2025 Jan 9;11:1486440. doi: 10.3389/fvets.2024.1486440 (PMC11754203; doi:10.3389/fvets.2024.1486440)
Supplement: Supplementary file 1 [file Data_Sheet_1.PDF]

Supplementary Material

1.1 Supplementary tables 1

1.2 Supplementary Figures

Figure s1

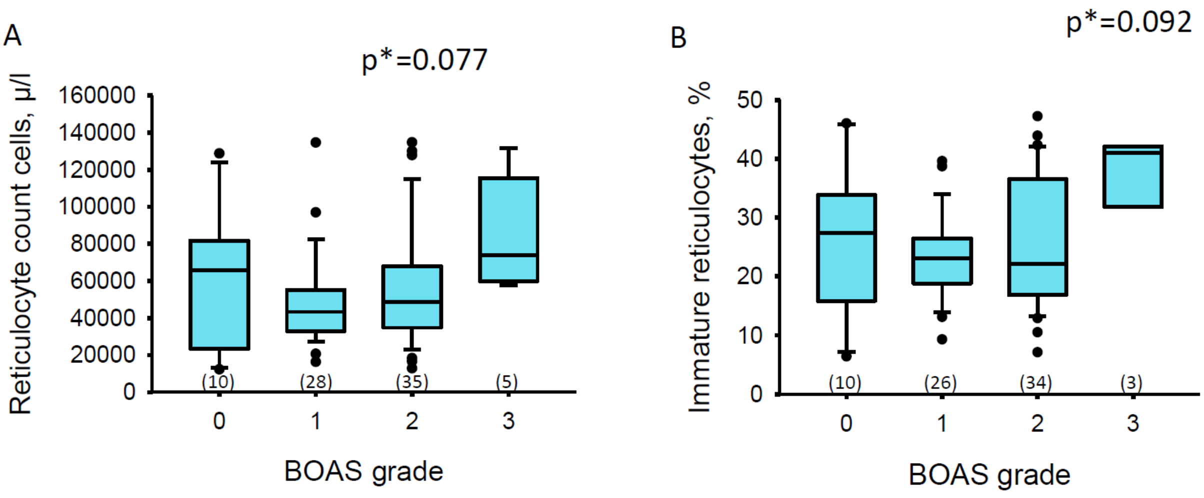

Supplementary Figure s1. Figure s1. Reticulocyte counts (A) and the abundance of immature reticulocytes (B) in blood of dogs with BOAS grades 0 to 3. Here and below numbers in brackets are for the numbers of dogs in each group. p* is a result of Kruskal-Wallis One Way Analysis of Variance (ANOVA) on Ranks as Shapiro-Wilk normality test failed.

Figure s2

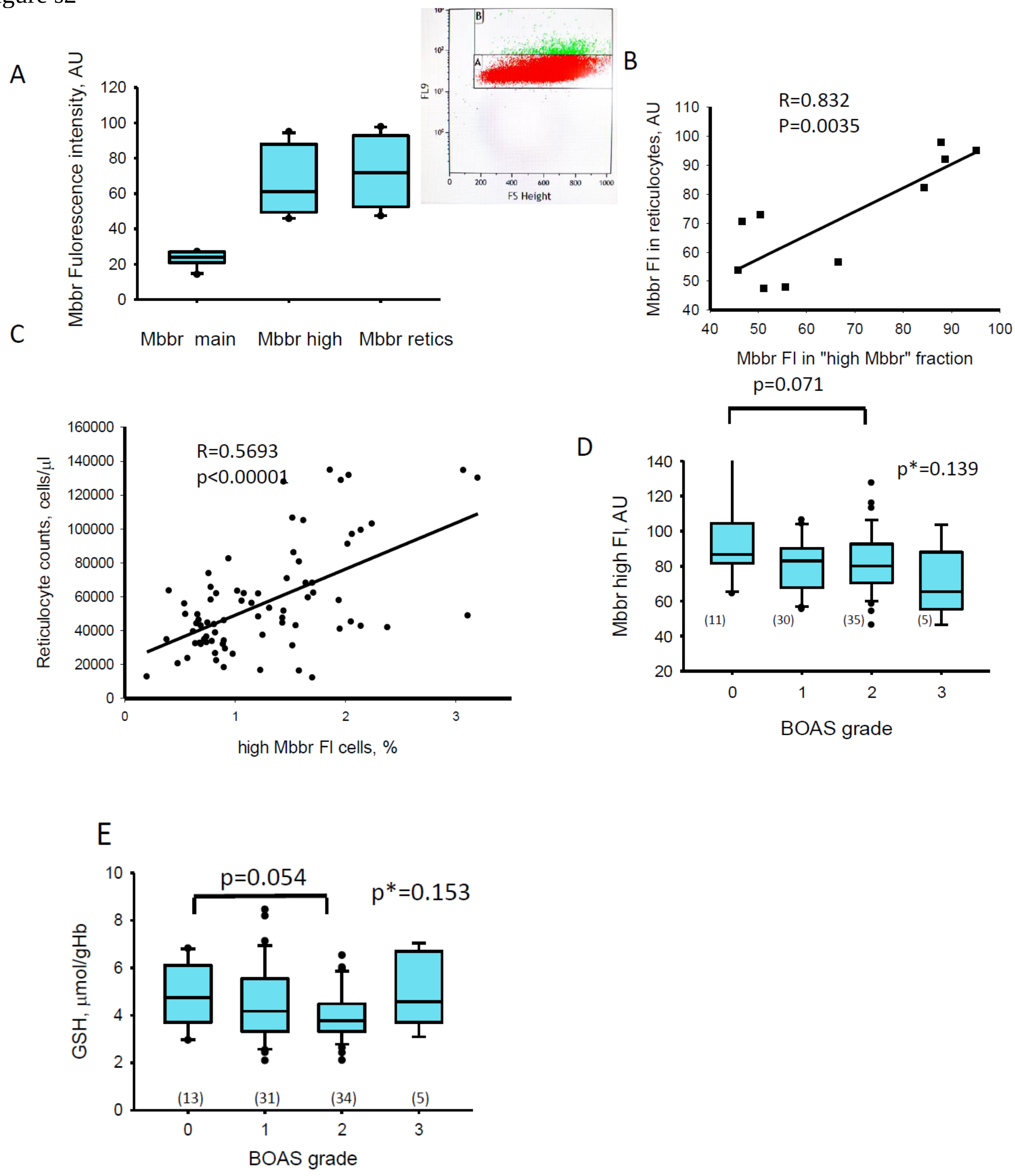
A
Mbbr Fulorescence intensity, AU
Mbbr main
Mbbr high
Mbbr retics
FL9
FS Height
B
R=0.832
P=0.0035
Mbbr FI in reticulocytes, AU
Mbbr FI in "high Mbbr" fraction
C
R=0.5693
p<0.00001
Reticulocyte counts, cells/μl
high Mbbr FI cells, %
D
p=0.071
p*=0.139
Mbbr high FI, AU
(11)
(30)
(35)
(5)
BOAS grade
E
p=0.054
p*=0.153
GSH, μmol/gHb
(13)
(31)
(34)
(5)
BOAS grade

Supplementary Figure s2. Redox state of reticulocytes vs bulk RBC population in dogs with BOAS grades 0-3. (A) Mbbr fluorescence signal as a marker of reduced protein and non-protein thiols in bulk RBC population (Mbbr main) and reticulocytes (Mbbr retics), as well as in the cells forming "high Mbbr fluorescence intensity cells" population (Mbbr high). Gating for "high Mbbr fluorescence intensity cells" is exemplified in the inset. Cells in the B gate are mainly reticulocytes. (B) Association between the fluorescence intensity in cells in the "high Mbbr fluorescence intensity cells" and reticulocytes, (C) Association between the reticulocyte counts and the abundance of cells in the "high Mbbr fluorescence intensity cells" fraction, (D) Fluorescence intensity of the cells in"high Mbbr fluorescence intensity cells" fraction of dogs with BOAS severity grades 0-3, (E) Intraerythrocytic reduced glutathione (GSH) content as a function of BOAS severity grade. Numbers in brackets denote the numbers of dogs in each group. p* stands for the One Way ANOVA (A, E), and One Way ANOVA on Ranks (D), p – for the unpaired Student's t-test.

Figure s3

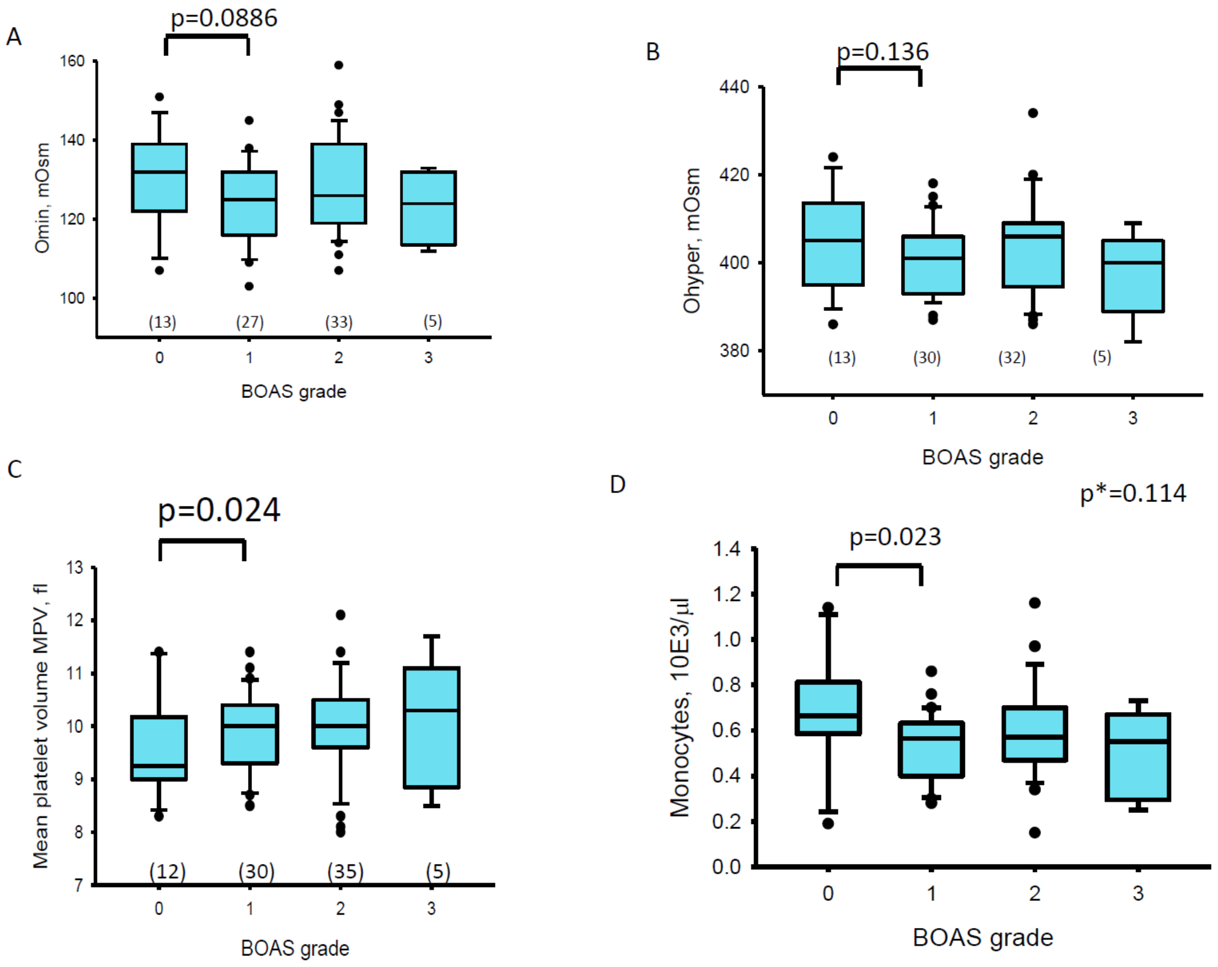

Supplementary Figure s3. Blood parameters showing potential association with BOAS grade. RBC osmotic stability was measured as minimal tolerated osmolarity Omin (A) and hydration state of RBCs detected as half-maximal tolerated osmolarity Ohyper, (C) mean platelet volume, and (D) monocyte counts. p* stands for the One Way ANOVA, p – for the unpaired Student’s t-test.

Figure s4

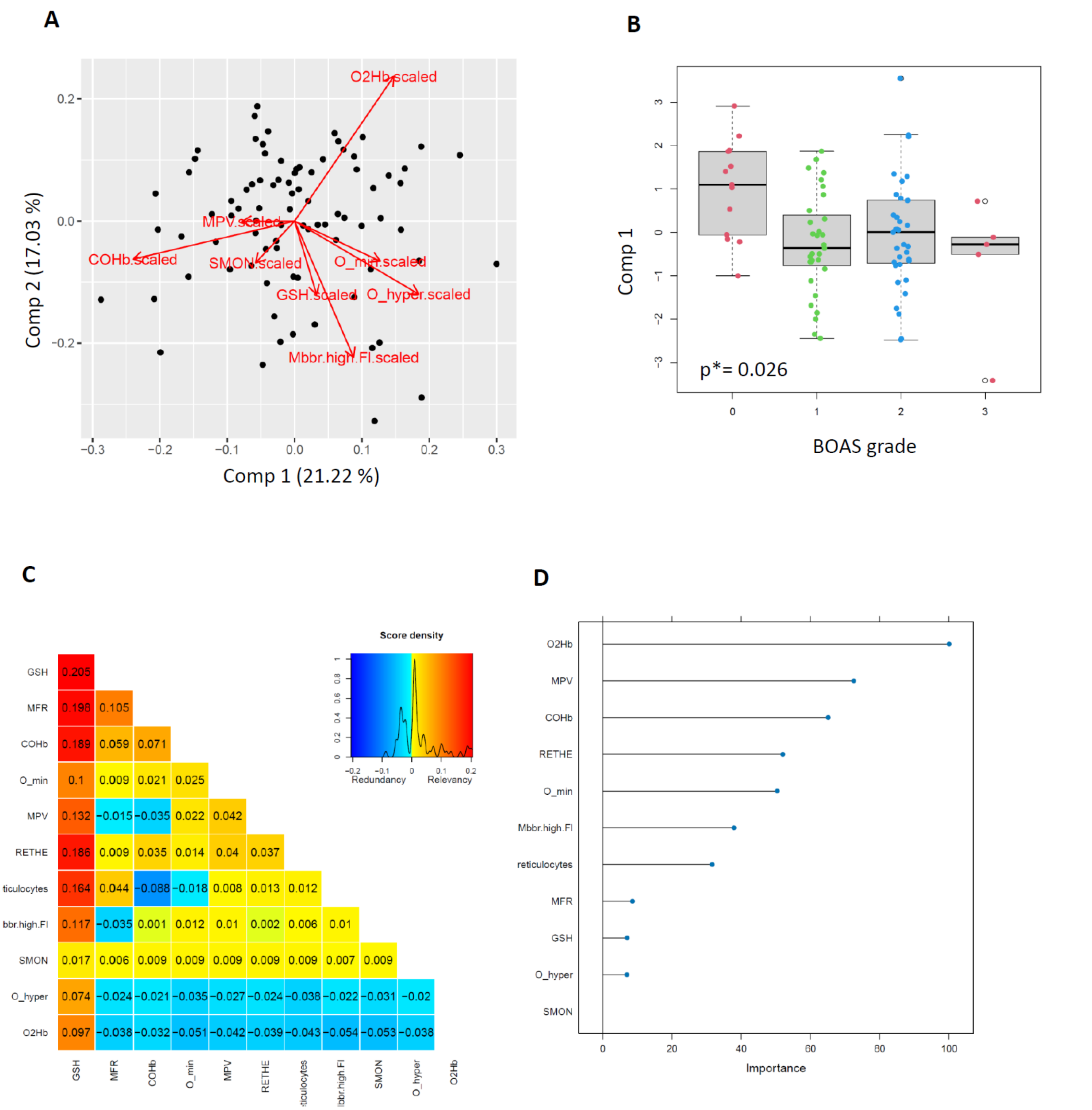

Supplementary Figure s4. Results of principal component analysis for the following collection of parameters: O2Hb, COHb, Mbbr fluorescence intensity of reticulocytes (Mbbr.high FI), GSH, O_min, O_hyper, mean platelet volume (MPV), monocyte counts (SMON). Principal component 1 was mainly dependent on COHb (-0.63), O_2Hb(0.39), O_hyper (0.48), O_min (0.33), Mbbr.high FI (0.22), other parameters contributed less into this component. (B) Association between principal component 1 and BOAS severity grade. p* is a result of the One Way ANOVA, (C) *varrank* and (D) *caret* algorithms applied to predict the association between the following blood parameters and

BOAS severity grades: O_2Hb, COHb, reticulocyte count, reticulocyte hemoglobin content (RETHE), Mbbr.high FI, GSH, medium fluorescence reticulocytes (MFR), SMON, MPV, O_min, and O_hyper.

1.3 Supplementary statistical analsis data

ET and Temperature differences

(for raw datasets see DOI 10.5281/zenodo.13358272)

1.4 Exploratory Data Analysis

```
# load data
  etdata <- read.csv("ET_temp_grade_v4.csv", sep=";")
  View(etdata)

# check for extreme an missing values (NAs)
  summary(etdata)

##      dog              temp_5min_pre   temp_5min_post   grade_5min
##  Length:84          Min.   :36.60   Min.   :37.00   Length:84
##  Class :character   1st Qu.:37.80   1st Qu.:37.80   Class :character
##  Mode  :character   Median :38.00   Median :38.10   Mode  :character
##                     Mean   :37.98   Mean   :38.08
##                     3rd Qu.:38.20   3rd Qu.:38.20
##                     Max.   :38.80   Max.   :39.10
##                     NA's   :9       NA's   :9
##  temp_3min_pre   temp_3min_post    grade_3min    temp_3min_diff
##  Min.   :36.60   Min.   :36.60   Min.   :0.000   Min.   :-0.6000
##  1st Qu.:37.92   1st Qu.:38.00   1st Qu.:1.000   1st Qu.: 0.0000
##  Median :38.10   Median :38.30   Median :1.000   Median : 0.2000
##  Mean   :38.11   Mean   :38.24   Mean   :1.381   Mean   : 0.1257
##  3rd Qu.:38.40   3rd Qu.:38.50   3rd Qu.:2.000   3rd Qu.: 0.2000
##  Max.   :39.00   Max.   :39.30   Max.   :3.000   Max.   : 1.1000
##  NA's   :10      NA's   :10                      NA's   :10
##  temp_5min_diff
##  Min.   :-0.60000
##  1st Qu.:-0.10000
##  Median : 0.20000
##  Mean   : 0.09867
##  3rd Qu.: 0.30000
##  Max.   : 0.80000
##  NA's   :9

# transform BOAS grades walking test into numbers
etdata$grade_5min[which(etdata$grade_5min=="Grad 0")] <- 0
etdata$grade_5min[which(etdata$grade_5min=="Grad 1")] <- 1
etdata$grade_5min[which(etdata$grade_5min=="Grad 2")] <- 2
etdata$grade_5min[which(etdata$grade_5min=="Grad 3")] <- 3
# str(etdata)
etdata$grade_5min <- as.numeric(etdata$grade_5min)
```

```r
# BOAS grades walking test
  walking_grades <- table(etdata$grade_5min)
  walking_grades

##
##  0  1  2  3
## 22 30 28  4

 # Barplot BOAS grade trotting test
  trotting_grades <- table(etdata$grade_3min)
  trotting_grades

##
##  0  1  2  3
## 13 31 35  5

# figure BOAS grades
par(mfrow = c(1,2))
barplot(walking_grades, ylab = "number of dogs", xlab = "BOAS grade", main = "Wa
lking Test", ylim = c(0, 35))
barplot(trotting_grades, ylab = "number of dogs", xlab = "BOAS grade",main = "Tr
otting Test")
```

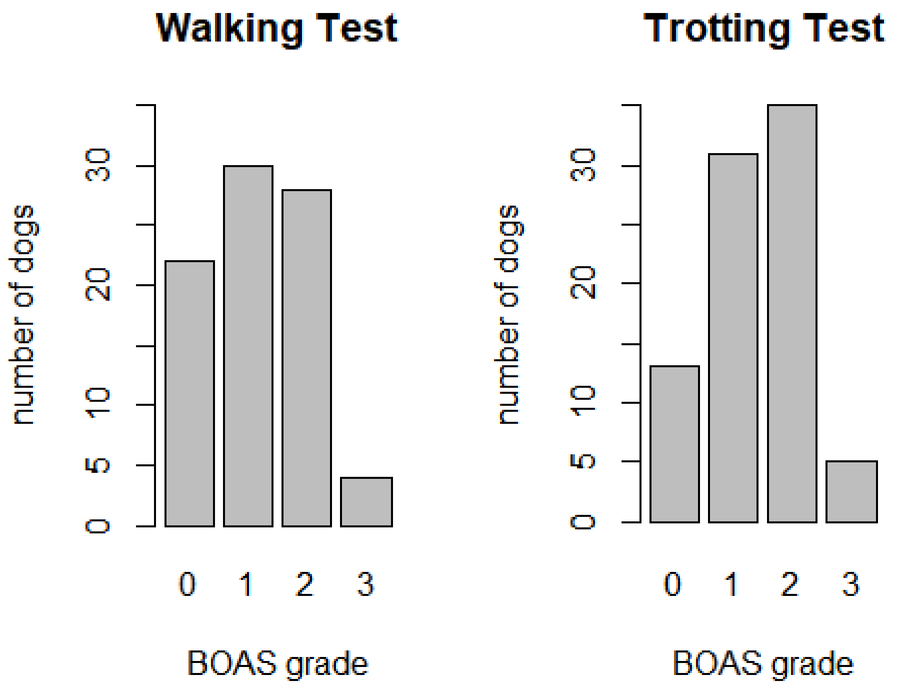


```r
# figures temperature difference by BOAS grade
par(mfrow = c(1,2))
plot(etdata$temp_5min_diff,etdata$grade_5min, main = "Walking Test", xlab = "Tem
perature Difference °C", ylab = "BOAS grade walking test", xlim = c(-0.8, 1.2),
yaxp = c(0, 3, 3), pch =16, col = etdata$grade_5min +1)
```

```
plot(etdata$temp_3min_diff, etdata$grade_3min,  main = "Trotting Test", xlab = "
Temperature Difference °C", ylab = "BOAS grade trotting test",xlim = c(-0.8, 1.2
), yaxp = c(0, 3, 3), pch =16, col = etdata$grade_3min +1)
```

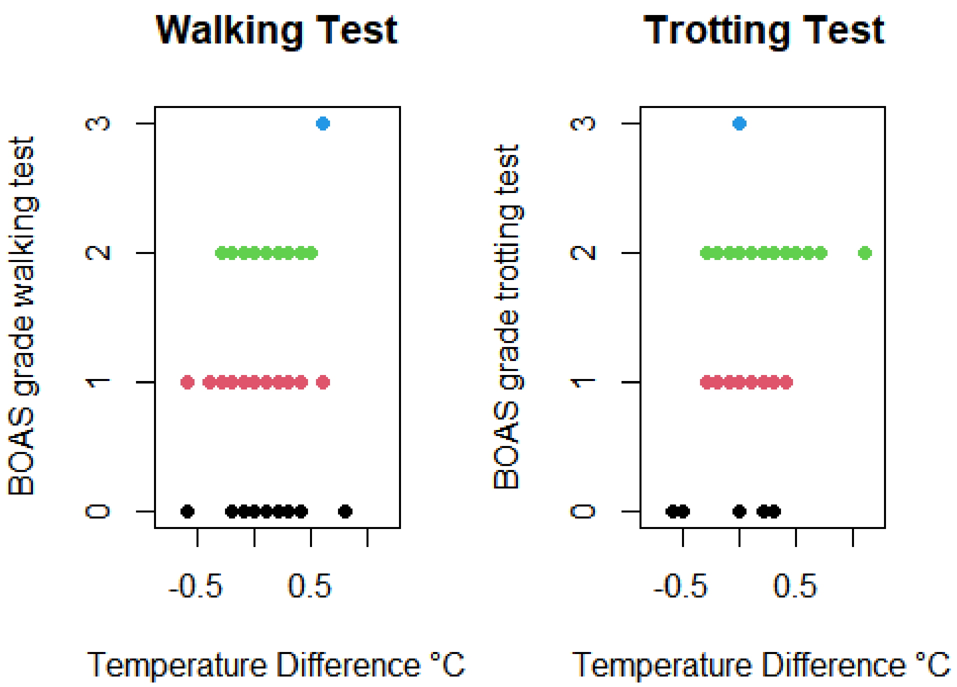

1.5 Comparison BOAS grades Walking vs. Trotting Test

```
# Walking Test lower grade
  sum(etdata$grade_5min < etdata$grade_3min) # 18 dogs lower grade in walking te
st

## [1] 18

  etdata$dog[which(etdata$grade_5min<etdata$grade_3min)]

##  [1] "BJ63" "CB71" "GA43" "JW94" "MO24" "QJ02" "RK44" "SF76" "TR54" "TU55"
## [11] "TV31" "VM46" "WF19" "XH31" "XW48" "YN33" "YV05" "ZG59"

# Walking Test higher grade
  sum(etdata$grade_5min > etdata$grade_3min) # 4 dogs

## [1] 4

  etdata$dog[which(etdata$grade_5min>etdata$grade_3min)]

## [1] "DM72" "JU09" "RH36" "TF71"
```

```
# Absolute difference of grades
  etdata$trot_minus_walk <- etdata$grade_3min - etdata$grade_5min
  etdata$dog[which(etdata$trot_minus_walk > 1)] # 3 dogs

## [1] "CB71" "VM46" "WF19"

  etdata$dog[which(etdata$trot_minus_walk < -1)] # 0 dogs

## character(0)

  differing_grades <- etdata[which(etdata$trot_minus_walk != 0), ]
  View(differing_grades)
```

1.6 ANOVA

Does it even make sense to perform ANOVAs with such massive overlap of the distributions???

Well... I do it for completeness.

Since only one dog per test is a grade 3 AND has temperature measurements, **grade 3** is **not considered in either ANOVA.**

```
# check how many dogs of grade 3 have temperature measurements
etdata$dog[which(etdata$grade_5min == 3 & etdata$temp_5min_diff != "NA")] # DM72

## [1] "DM72"

etdata$dog[which(etdata$grade_3min == 3 & etdata$temp_3min_diff != "NA")] # VM46

## [1] "VM46"

# create cleaned dataset for ANOVA alking test
which(etdata$grade_5min == 3 & etdata$temp_5min_diff != "NA")

## [1] 14

walkANOV <- etdata[-14,]
# ANVOA walking test
model1 <- lm(temp_5min_diff ~ grade_5min, data = walkANOV)
par(mfrow = c(2,2))
plot(model1)          # assumptions fulfilled
```

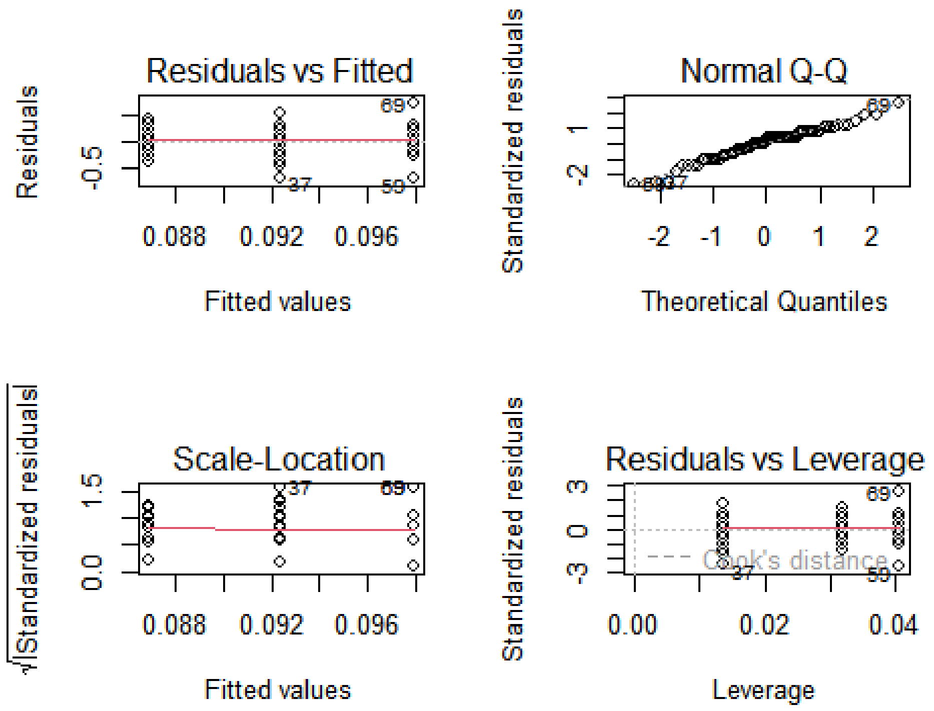


```
summary(aov(model1)) # not significant

##             Df Sum Sq Mean Sq F value Pr(>F)
## grade_5min   1  0.001 0.00133   0.017  0.896
## Residuals   72  5.614 0.07797
## 9 Beobachtungen als fehlend gelöscht

# create cleaned dataset for ANOVA trotting tes
which(etdata$grade_3min == 3 & etdata$temp_3min_diff != "NA")

## [1] 66

trotANOV <- etdata[-66,]
# ANVOA trotting test
model2 <- lm(temp_3min_diff ~ grade_3min, data = trotANOV)
plot(model2)          # assumptions fulfilled (normality within tolerance)
```

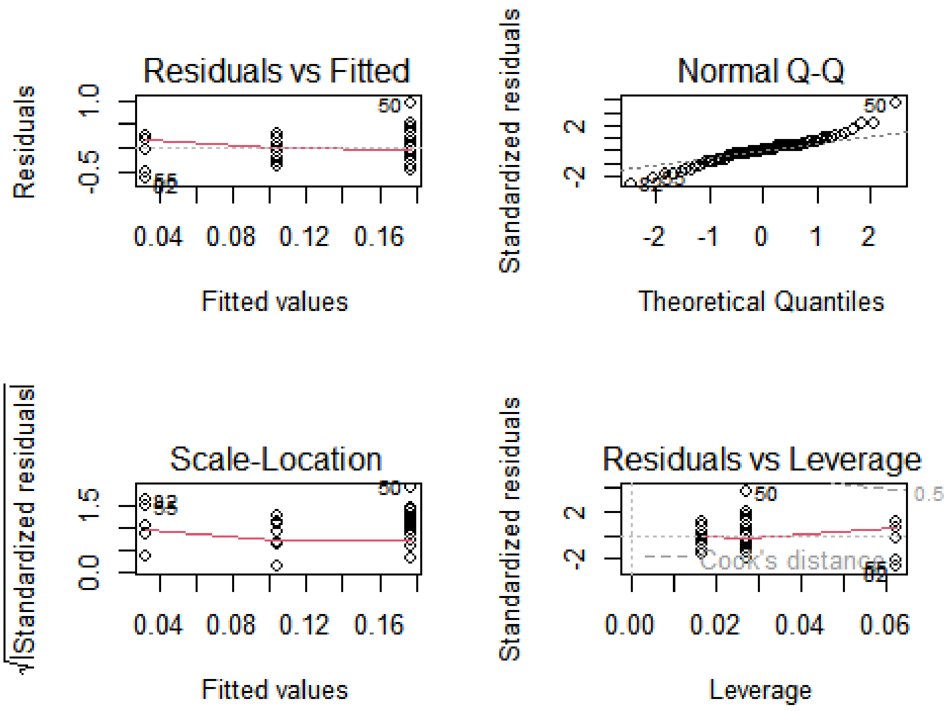


```
summary(aov(model2)) # not significant

##              Df Sum Sq Mean Sq F value Pr(>F)
## grade_3min    1  0.185 0.18472   2.838 0.0964 .
## Residuals    71  4.620 0.06508
## ---
## Signif. codes:  0 '***' 0.001 '**' 0.01 '*' 0.05 '.' 0.1 ' ' 1
## 10 Beobachtungen als fehlend gelöscht
```
